# Do we still need to screen our patients?—Orthopaedic scoring based on motion tracking

**DOI:** 10.1007/s00264-022-05670-0

**Published:** 2023-01-10

**Authors:** Dominik Raab, Falko Heitzer, Jin Cheng Liaw, Katharina Müller, Lina Weber, Francisco Geu Flores, Andrés Kecskeméthy, Constantin Mayer, Marcus Jäger

**Affiliations:** 1 Chair of Mechanics and Robotics, University of Duisburg-Essen, Lotharstraße 1, 47057 Duisburg, Germany; 2 Chair of Orthopaedics and Trauma Surgery, University of Duisburg-Essen, Essen, Germany; 3 Department of Orthopaedics, Trauma and Reconstructive Surgery, St. Marien-Hospital Mülheim an der Ruhr, Mülheim an der Ruhr, Germany

**Keywords:** Harris Hip Score, Knee Society Score, Patient-reported outcome measures, Instrumental gait analysis, Gait feature extraction

## Abstract

**Purpose:**

Orthopaedic scores are essential for the clinical assessment of movement disorders but require an experienced clinician for the manual scoring. Wearable systems are taking root in the medical field and offer a possibility for the convenient collection of motion tracking data. The purpose of this work is to demonstrate the feasibility of automated orthopaedic scorings based on motion tracking data using the Harris Hip Score and the Knee Society Score as examples.

**Methods:**

Seventy-eight patients received a clinical examination and an instrumental gait analysis after hip or knee arthroplasty. Seven hundred forty-four gait features were extracted from each patient’s representative gait cycle. For each score, a hierarchical multiple regression analysis was conducted with a subsequent tenfold cross-validation. A data split of 70%/30% was applied for training/testing.

**Results:**

Both scores can be reproduced with excellent coefficients of determination *R*^2^ for training, testing and cross-validation by applying regression models based on four to six features from instrumental gait analysis as well as the patient-reported parameter ‘pain’ as an offset factor.

**Conclusion:**

Computing established orthopaedic scores based on motion tracking data yields an automated evaluation of a joint function at the hip and knee which is suitable for direct clinical interpretation. In combination with novel technologies for wearable data collection, these computations can support healthcare staff with objective and telemedical applicable scorings for a large number of patients without the need for trained clinicians.

## Introduction

Clinical scores are the most commonly used tool for the systematic evaluation of orthopaedic movement disorders [1. –4. ]. Most of them are based on clinical examination and patient-reported information. This allows easy applications within few minutes by a trained clinician [5. , 6. ]. However, due to the economic burden in healthcare systems and staff shortages [7. ], it is becoming increasingly challenging to maintain experts’ scorings for a rapidly rising number of patients [8. ]. Furthermore, systems for wearable motion tracking are on the rise and transforming approaches for the assessment of patients’ health [9. ]. Wearables can provide objective and high-resolution motion data in a clinical setting as well as continuous data in a habitual setting, providing information that cannot be detected by human assessment [9. –11. ]. Therefore, we hypothesise that motion tracking offers an innovative approach for orthopaedic scoring of impaired movement such as osteoarthritis or after total joint replacement. These systems may not only reduce clinical staffs’ workload by providing an automated assessment, but also provide further insights into the following rehabilitation process. Moreover, motion tracking-based scoring allows an objective, observer-independent assessment. However, up to date, this raw motion data is excessive and possesses a strong mathematical character. For this reason, the data is not suitable for daily orthopaedic practice and requires extended training for the interpretation.

In this work, we demonstrate the feasibility of automated clinical scoring by motion tracking data. We chose the Harris Hip Score (HHS) [12. , 13. ] and the Knee Society Score (KSS) [3. , 14. ] since these scores are well established, have shown high validity and reliability and are emphasised to be used by national registries as patient-reported outcome measures [1. , 14. , 15. ]. Since a broad spectrum of wearables is available, which are based on competing measuring principles (e.g. inertial measurement units, radar-based devices, optical systems) [16. , 17. ] and feature a broad range of system parameters, the authors decided to apply a high-resolution 3D gait analysis system for this study in order to investigate the feasibility independently of a specific wearable system.

## Materials and methods

### Participants

The inclusion criteria for the orthopaedic patients were (1) condition after total hip or knee replacement, (2) three to six days recovery time after surgery, (3) adequate recovery status evaluated by the orthopaedic surgeon, (4) the ability to walk at least ten metres without the assistance of a therapist, (5) age ≥ 18 years and (6) written informed consent. The exclusion criteria were (1) insufficient post-operative recovery, (2) a generally inadequate state of health (evaluated by the attending physical therapist) and (3) participation in another study.

### Data collection

Patients underwent a clinical examination directly prior to an instrumental gait analysis. During clinical examination, the patients were assessed with the HHS or the KSS-knee score (KSS-ks) by an experienced clinician. The gait analysis was performed with a Vicon 3D motion capture system with six tracking cameras (100 Hz, Oxford Metrics Ltd., England). A full-body plug-in gait marker set was attached to the patients’ legs, arms, trunk and head [18. ]. All subjects walked barefoot at a self-selected speed along a ten metre walkway. For each subject, data of at least four trials was collected.

### Feature extraction from gait analysis data

**Fig. 1:**
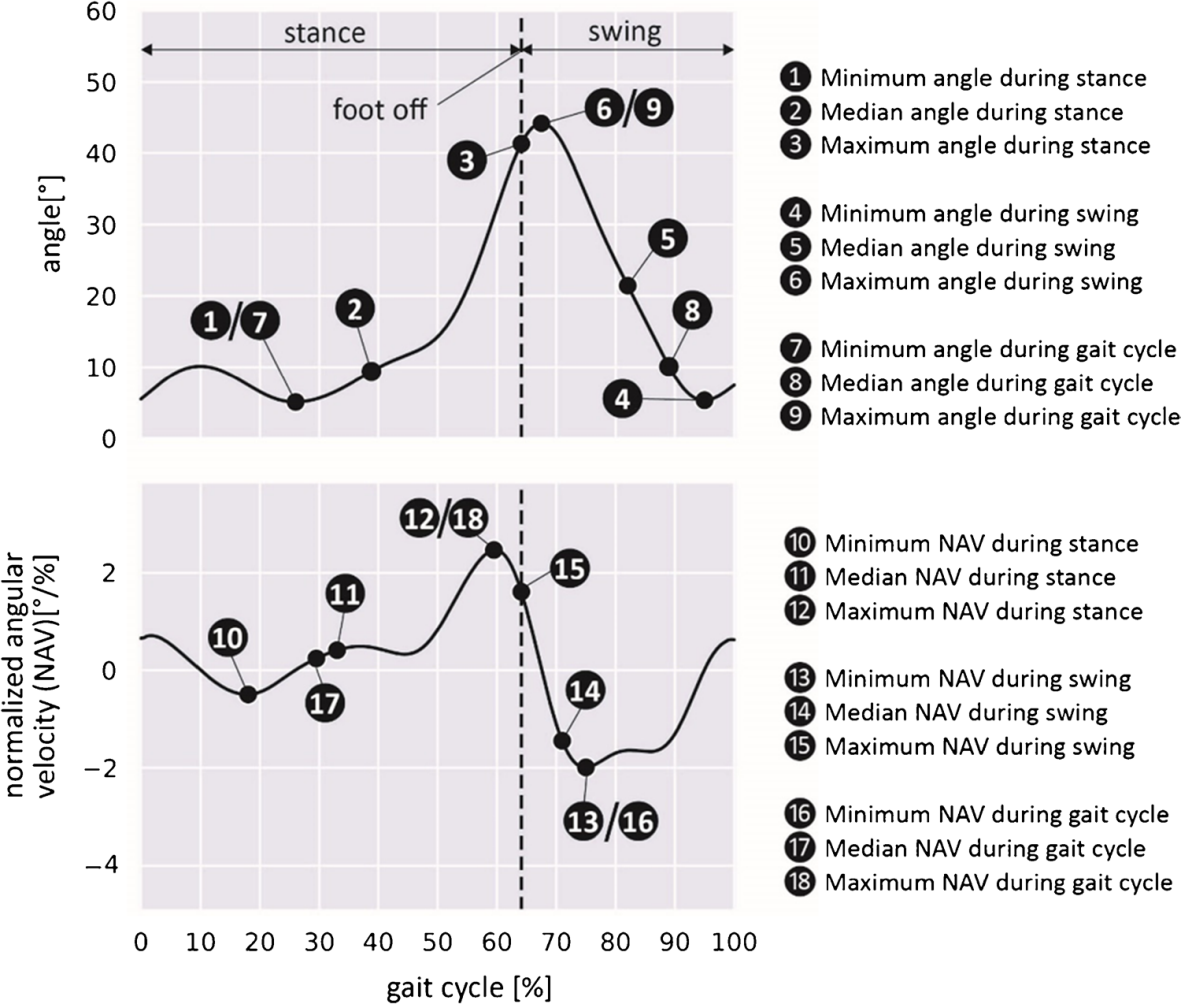
Characteristics of movement illustrated at the angle ‘knee flexion/extension of the affected side’

For each subject, a representative gait cycle was selected according to Schweizer et al. [19. ]. After time normalisation of the representative gait cycles to 100% and designation of the affected and the contralateral side according to the case report file, 372 gait features were extracted for each side, leading to 744 features for each subject. The features consist of 60 gait parameters and 684 characteristics of the movement of main joints and segments of the locomotor system. The gait parameters include 14 standard parameters of instrumental gait analysis which are listed in Table 1 and which were defined according to Hollmann et al. [20. ]. Sixteen additional parameters were derived by the evaluation of the start and duration of nine distinguished functional phases of the gait cycle (stance, swing and seven gait phases according to Perry [21. ]: loading response to terminal swing). Since the phases loading response and stance always start at 0% of the gait cycle, the start of these phases is not included as parameters. Furthermore, for a nuanced evaluation of the patients’ gait, the characteristics of 19 physiological angles of the locomotor system are examined (see Table 1). For each angle, 18 features are extracted, consisting of the minimum, median and maximum of the physiological angles and their corresponding normalised angular velocities (NAVs) during stance, swing and the whole gait cycle (see Fig. 1). The use of NAVs for feature extraction is a novel concept in gait analysis and is introduced here with the purpose of characterising the dynamics of joint movement, which the authors consider essential for the assessment of the joint function. The NAV describes the slope of the tangent to the plot of the corresponding angle against the gait cycle progress (0 to 100%) and has the unit °/%. The NAVs are obtained with the software MobileBody (ITBB GmbH, Germany) by interpolating the position and orientation of each body segment with B-splines of degree five, using the gait cycle progress as the B-spline parameter and quaternions to parametrise orientation, and then mapping the B-spline derivatives to the corresponding angular velocities.

**Table 1:** Overview of the features extracted from gait analysis data

Gait parameters (*n* = 60: 30 parameters * 2 sides)	Characteristics of movement (*n* = 684: 19 angles * 18 characteristics * 2 sides)	
Standard parameters (14 × 2 sides): • Gait speed (km/h) • Cadence (steps/min) • Stride time, step time, stance time, swing time, single support time, double support time (s) • Single, double support duration (%) • Step length, stride length, step width (m) • Limp index (–)	Pelvis	Tilt, obliquity, rotation
	Hip joint	Flexion/extension, adduction/abduction
	Knee joint	Flexion/extension
	Ankle joint	Dorsiflexion/plantarflexion, rotation
	Foot	Progression
	Thorax	Tilt, side tilt, rotation
	Shoulder joint	Flexion/extension, adduction/abduction, rotation
Phase parameters (16 × 2 sides): • Start (%) of swing and 6 Perry phases • Duration (%) of stance, swing and 7 Perry phases	Elbow joint	Flexion/extension
	Forearm	Pronation/supination
	Wrist joint	Dorsi-/plantarflexion, deviation

### Statistical analysis

A hierarchical stepwise regression analysis was conducted for each score with a random data split of 70% for training and 30% for testing. For training, a minimal sample size of 23 participants was determined with GPower 3.1.9.7 [22. ] by calculating the number of participants necessary to reach a statistical power of 75% and a large effect size at a significance level of 0.05. Input data were the 744 features, grouped in order of descending relevance based on medical background knowledge to support the data-driven predictor selection. The grouped features were added to the model by applying thresholds for *p* values (*p*_enter_ = 0.05, *p*_remove_ = 0.10), leading to several regression models. In order to avoid overfitting, the maximum number of predictors for a linear regression model obtained with the given sample size was calculated according to Tabachnick and Fidell [23. ]. Finally, the model with the best goodness of fit, in compliance with the maximal number of predictors, was chosen and tested with the remaining 30% of the participants. Afterwards, a tenfold cross-validation was conducted in order to approximate an average goodness of fit for the chosen regression models. All statistics were calculated using IBM SPSS Statistics 27. A critical level of *p* < 0.05 was considered significant for all statistics. According to Cohen’s recommendations, the threshold for a high goodness of fit was set at *R*^2^ ≥ 0.26 [24. ].

## Results

### Participants

The modelling is based on 78 patients, from which 44 persons were assessed with the HHS and 34 with the KSS-ks. Participants’ characteristics are presented in Table 2.

**Table 2:** Participants

	HHS	KSS-ks
Sample size	44	34
Medical treatment	Total hip replacement	Total knee arthroplasty/partial knee arthroplasty
Age (years): mean (SD)	66.36 (9.95)	67.00 (8.53)
Gender: male/female	25/19	14/20
Affected side: left/right/both	19/25/0	15/19/0
Walking aid: none/one side/both sides	6/1/37	8/1/25
Score: mean (minimum–maximum)	61 (30–92)	65 (28–88)

### Grouping of input features

For the data-driven predictor selection, the input features were grouped in order of descending relevance: the parameters of the affected joint were used at the first regression stage (hip for HHS and knee for KSS-ks). Due to the high range of motion in the knee and hip joints during physiological gait, these joints were selected as the second regression stage (knee for HHS and hip for KSS-ks). Since the angles of the pelvis and foot progression are essential for the physiological forward locomotion, the associated features were included in the third stage. Next, the ankle, thorax and arm features were assigned, because these features respond to the pathology in the hip or knee joint in descending order. Finally, as the last regression stage, the gait parameters were added, as these features are not directly connected with the clinical examination.

### Linear regression

**Fig. 2:**
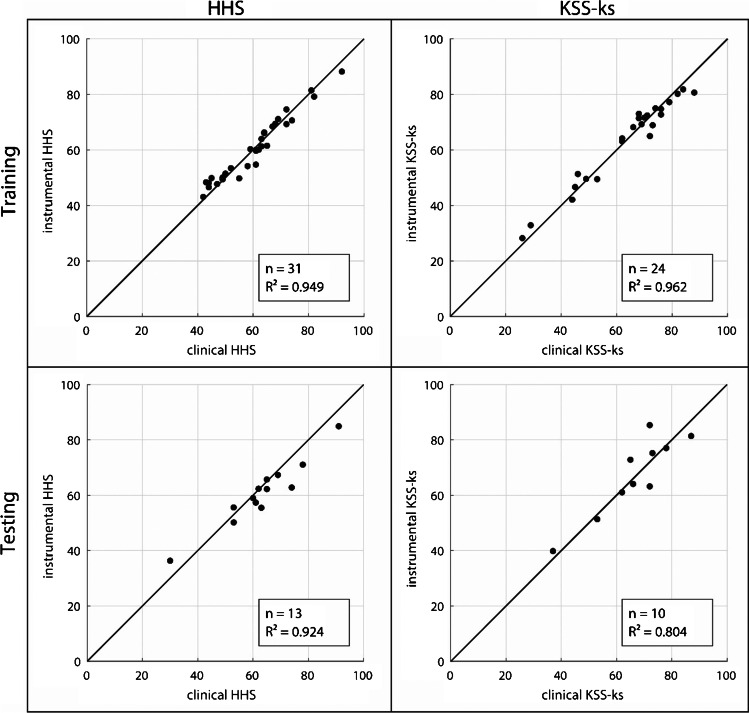
Scatterplots and performance characteristics of the training and testing of the regression models

Figure 2 shows the performance parameters ‘goodness of fit’ *R*^2^ for the training (upper row) and testing (lower row) of the chosen regression models for the HHS (left column) and the KSS-ks (right column) along with scatterplots visualising the relation of the clinical scorings and the instrumental scores. Please note that the black line inclined at a 45° angle denotes a total agreement. The results of the tenfold cross-validation accompany these findings with $\overline R_{\mathrm{HHS}}^2=0.894\;\mathrm{and}\;\overline R_{\mathrm{KSS}-\mathrm{ks}}^2=0.864$.

The model predictors with their relative weighting are shown in Table 3. Please note that due to difficulties in modelling the individual pain perception, the regression models add the patient-reported parameter ‘pain’ as an offset factor. Both regression models are absent of multicollinearity, no outliers were observed and the residuals are normally distributed and homoscedastic.

**Table 3:** Model predictors for HHS and KSS-ks

Score	No	Angle	Side	Plot	Phase	Feature	Coeff	Sig
HHS	1	Hip—flex/ext	Affected	NAV	Swing	Median	0.076	0.021
	2	Hip—add/abd	Contralat	NAV	Stance	Max	0.075	0.016
	3	Pelvis—tilt	Contralat	NAV	Gait cycle	Median	− 0.396	0.029
	4	Knee—flex/ext	Affected	NAV	Gait cycle	Median	0.232	0.001
	5	Knee—flex/ext	Affected	NAV	Stance	Max	0.043	0.006
	6	Wrist—deviation	Contralat	Angle	Swing	Median	− 0.235	0.000
	7	Constant	–				25.786	0.000
	8	Pain (offset)	–				1.000	–
KSS-ks	1	Knee—flex/ext	Affected	NAV	Stance	Median	− 0.283	0.002
	2	Hip—add/abd	Affected	NAV	Gait cycle	Max	0.121	0.007
	3	Foot progression	Contralat	Angle	Stance	Median	0.561	0.002
	4	Wrist—dorsi-/plantarflex	Affected	NAV	Stance	Max	− 0.267	0.000
	5	Constant	–				39.680	0.000
	6	Pain (offset)	–				1.000	–

Our data show that the regression models applied are robust and are able to generate reliable HHS and KSS-ks score values in patients after total hip or knee replacement by motion tracking.

## Discussion

The HHS and KSS-ks regression models are characterised by excellent coefficients of determination (HHS: training *R*^2^ = 0.95, testing *R*^2^ = 0.92, cross-validation $\overline{R }$ ^2^ = 0.89; KSS-ks: training *R*^2^ = 0.96, testing *R*^2^ = 0.80, cross-validation $\overline{R }$ ^2^ = 0.86). This suggests robust models for deriving the orthopaedic scores from gait data. Furthermore, the identified predictors are clinically plausible and can be explained as follows:

HHS-1: hip flexion/extension:

- The magnitude and speed of the angular change of the hip flexion during swing are a key indicator of the functionality of the hip joint [25. ].

HHS-2: hip adduction/abduction:

- Restricted gluteal function (especially Trendelenburg gait) [26. ] can be present postoperatively due to the surgical approach and allows conclusions about the muscular capacity of the patient [27. ].

HHS-3: pelvis tilt:

- From the tilting of the pelvis throughout the gait cycle, the use of supports can be inferred. The increased pelvic tilting ventrally represents the shift of the body’s centre of gravity across the body axis. This forward shift is made possible with the supporting walking aid only.

HHS-4 and HHS-5: knee flexion/extension:

- These parameters detect an early foot strike, an insufficient depth of hip flexion or a limp.
- Before the end of the stance phase, the swing phase is initiated by a maximum speed during angular change (deficits in muscle activity and joint motion range can be determined).

HHS-6: wrist deviation:

- Increased radial deviation during the swing phase reflects relief of the stance leg via support on walking aids.

KSS-ks-1: knee flexion/extension:

- Indicator for intact/restored knee kinematics.
- Full extension is often avoided due to chronic or postoperative effusions and impairment of the extensor mechanism due to the surgical approach [28. ].

KSS-ks-2: hip adduction/abduction:

- Greater internal hip abduction moment was proven to protect against medial femorotibial osteoarthritis progression [29. , 30. ].
- Valgus instability of the knee is compensated in patients gait by adduction of the hip joint and known to be a clinically relevant factor [31. ]

KSS-ks-3: foot progression:

- Deficits in the swing phase of the affected leg are compensated by external rotation of the contralateral foot [32. ].

KSS-ks-4: wrist dorsiflexion/plantarflexion:

- Unintentional dropping (dorsiflexion) of the wrists due to the load distribution by supporting walking aids in the stance phase (similar findings by Requejo et al.) [33. ].

### Normalised angular velocities

NAV-based features are very uncommon in gait analysis. To the best of the authors’ knowledge, only Granata et al. apply a similar concept so far [34. ]. The result that eight out of ten predictors are NAV features implies that these features contain relevant information which is not included in features extracted from physiological angles.

### Suitability for wearable data acquisition

The identified predictors (Table 3) are well suited for wearable data acquisition since they consist of kinematic parameters which can be derived from measurements with existing wearable motion tracking systems, such as IMU sensors [10. ]. Since the predictors focus on the pelvis, hip, knee, foot and wrist, a reduced body model is conceivable by removing the sensors at the head, trunk and upper arms. In this way, the time required for the measurements (approx. 8 min for placement and removal of full-body IMU sensors [35. , 36. ]) can be further reduced, promising a fast data collection and processing, comparable to the time expenditure of the equivalent clinical scoring but with no need for an expert clinician.

### Limitations

The modelling has three limitations. First, the regression models are based on postsurgical patients after total hip or knee replacement. Further studies are necessary to show if the regression models are applicable for generic orthopaedic patients or if the models have to be adjusted according to the clinical application. Second, the number of cases for training and testing is at the lower end of the statistical requirements for a regression analysis. However, the authors took this limitation already into account by constraining the number of predictors and by conducting a tenfold cross-validation in order to rule out overfitting. Third, the parameter ‘pain’ must be added as a manual offset factor. Nevertheless, since the assessment of the patient-reported outcome ‘pain’ consists of a single question, a corresponding query can be integrated into the measurement software with minimal effort.

## Conclusion

The orthopaedic scores HHS and KSS-ks can be reproduced very well by linear regression models based on four to six features from instrumental gait analysis and the patient-reported parameter ‘pain’ as an offset factor. This proves the feasibility of automated clinical scorings of orthopaedic movement disorders. While clinical scores are based on static examinations and subjective questionnaires, the instrumental scores are derived from reproducible and nuanced gait analysis features, which quantify dynamic aspects of the treated joint’s movement as well as compensatory movements of the whole body. In contrast to the existing gait analysis parameters, the instrumental scores are suitable for direct clinical application since they reproduce renowned clinical rating scales. In combination with existing technologies for wearable gait data collection, the instrumental HHS and KSS-ks promise a pragmatic evaluation of the joint function at the hip and knee. The instrumental scores may support healthcare staff by supplying automated and objective clinical scorings for a large number of patients without the need for trained clinicians. Furthermore, amid the ongoing development of novel technologies for gait data collection at home, they may as well contribute to establish a close-meshed telemedical feedback on patients’ health status in the near future, paving the way for novel applications in therapy management and personalised healthcare.

## Data Availability

Not applicable.
